# Development of an ELISA using the recombinant protein CP1957 of *Corynebacterium pseudotuberculosis *for diagnosis of caseous lymphadenitis in sheep

**DOI:** 10.1186/1753-6561-8-S4-P163

**Published:** 2014-10-01

**Authors:** Carlos Guilherme Rosa Reis, Henrique Ramos Angelo, Andréa de Fátima Silva Rezende, Alexandre Antunes Brum, Vasco Ariston de Carvalho Azevedo, Sibele Borsuk

**Affiliations:** 1 Research Laboratory for Infectious Diseases, Center of Technological Development, Biotechnology, Universidade Federal de Pelotas, Pelotas, RS, Brazil; 2 Laboratory of Cellular and Molecular Genetics, Department of General Biology, Universidade Federal de Minas Gerais, Belo Horizonte, MG, Brazil

## Background

Caseous lymphadenitis (CLA) is a disease caused by the bacteria *Corynebacterium pseudotuberculosis *which affects small ruminants such as sheeps and goats, leading to severe economic losses. The development of more sensitive and specific diagnoses showing effectiveness on asymptomatic animals is essential for disease's control. This study purposes the use of the recombinant protein CP1957 of *C. pseudotuberculosis *in indirect ELISA using sheep sera.

## Methods

The amplification of the *cp1002_1957 *gene was performed using the primers F5' CGCGGATCCGGGCCTCGCGACTGG 3' and R5' CCGGAATTCTTACCAGGCGTTCATAACGT 3'. The *cp1002_1957 *gene was cloned in the *Bam*HI e *Eco*RI sites of the pAE vector. The recombinant clones (pAE/1957) were characterized enzymatically and by DNA sequencing. *E. coli *BL21 Star cells were transformed with the pAE/1957 vector for the expression of rCP1957 protein, the induction was performed by the addition of IPTG 1mM to the culture media. The purification was realized by affinity chromatography on a Sepharose column loaded with nickel. The purity was determined using a 12% SDS-PAGE, and the concentration determined by a BCA kit. For indirect ELISA, the purified rCP1957 was utilized as antigen in a concentration of 1µg/mL. The sheep sera and the anti-sheep conjugated with peroxidase were used in 1:100 and 1:5000 dilutions, respectively. A total of 49 sheep sera were analyzed, where 14 were from asymptomatic animals and 35 were from negative animals. The sensitivity and specificity of ELISA-r1957 were analyzed on receiver operating characteristic (ROC).

## Results and conclusion

The statistical analysis of the ELISA-rCP1957 results presented sensitivity and specificity values of 92.9% e 85.7%, respectively, when the absorbance cutoff value of 0.063 was used. Thus, we can conclude that the developed ELISA, using the recombinant protein CP1957 can be used for the CLA diagnoses with good levels of sensitivity and specificity.
